# Acute presentation of a solitary caecal diverticulum: a case report

**DOI:** 10.1186/1752-1947-1-129

**Published:** 2007-11-09

**Authors:** Ewen A Griffiths, Ravindra S Date

**Affiliations:** 1 Department of General Surgery, Blackpool Victoria Hospital, Blackpool Fylde and Wyre NHS Trust, Blackpool, FY2 8NR, UK; 2 Department of Surgery, Altnagelvin Area Hospital, Londonderry, Northern Ireland, BT47 6SB, UK

## Abstract

**Introduction:**

Solitary caecal diverticulitis is a rare cause of abdominal pain in Caucasian patients. The condition is often misdiagnosed and only correctly identified on exploration for suspected acute appendicitis. Our aim is to improve awareness of this condition amongst surgical trainees to ensure that its first encounter is not in the operating theatre. We review the role of pre-operative radiological imaging in this condition and the wide and controversial management options are also discussed.

**Case presentation:**

A 67 years old man was admitted with a 24 hour history of pain in right iliac fossa. A pre-operative diagnosis of acute appendicitis was made but at operation a 2.5 cm inflamed and gangrenous solitary diverticulum of caecum was found. This was treated by right hemicolectomy as there was the suspicion of underlying malignancy.

**Conclusion:**

Caecal diverticulitis, although rare in the Western population, should be considered in the differential diagnosis of patients complaining of right iliac fossa pain. The surgical approach should be tailored to the clinical scenario but may include conservative management, diverticulectomy, limited ileocaecal resection or right hemicoloectomy.

## Background

Right iliac fossa pain, nausea and vomiting are common symptoms that require acute surgical assessment. The differential diagnosis is vast and includes acute appendicitis, gastroenteritis, ureteric colic, ectopic pregnancy, rupture ovarian cyst, and pelvic inflammatory disease. Solitary caceal diverticulitis is a rare cause of abdominal pain in Western patients and is more common in the Oriental population [1-3]. The symptoms and signs of the disease closely mimic acute appendicitis [1,3,4]. As such the condition is often misdiagnosed and only correctly diagnosed on exploration for suspected acute appendicitis.

We describe a patient who was thought to have acute appendicitis pre-operatively, however at operation an inflamed and gangrenous solitary caecal diverticulum was found. We review the literature surrounding the role of pre-operative radiology in this condition and review the controversial surgical management. As the condition is frequently misdiagnosed and often mistreated, our aim is to improve awareness of this condition amongst surgical trainees to ensure it is appropriately treated when encountered unexpectedly.

## Case report

A 67 years old man was admitted with a 24 hour history of pain in right iliac fossa. He was previously healthy and had no significant past medical history. He had no other symptoms. On examination, he was apyrexial and had localised guarding and peritonism in his right iliac fossa. His blood investigations, including full blood count, were normal. A provisional diagnosis of acute appendicitis was made.

**Figure 1:**
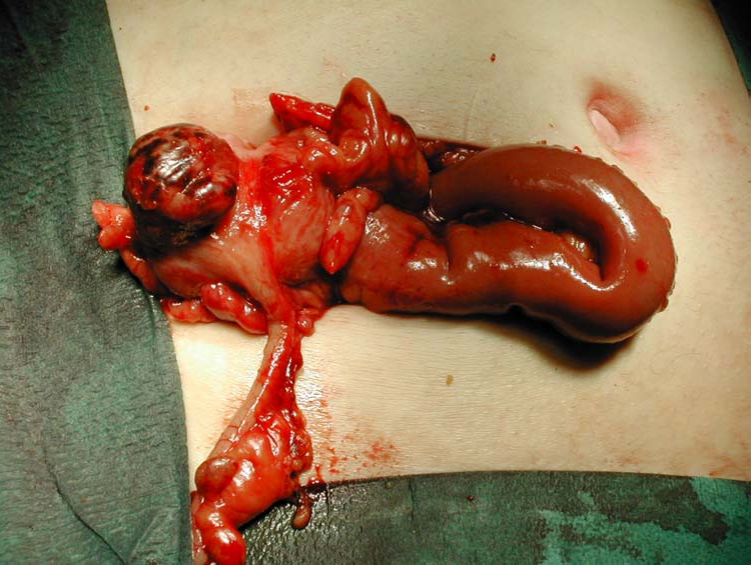
Intra-operative photograph shows the gangrenous caecal diverticulum with a normal appendix.

At operation, through a Lanz incision, the appendix was found to be normal. A 2.5 cm gangrenous solitary diverticulum of caecum was found (Figure 1).

The caecal wall near to the diverticulum was thickened and abnormal, raising the suspicion of underlying carcinoma. The wound was extended and a right hemicolectomy with appropriate cancer clearing lymphadenectomy was then performed. Histopathology of the resected right colon showed that the lesion was a solitary caecal diverticulum. The diverticula comprised all layers of the colonic wall indicating that it was a 'true' type. There were histological features of acute inflammation and gangrene, but no evidence of malignancy. The patient's post-operative course was uneventful and he was discharged 7 days after admission. He remained well when reviewed in the outpatient clinic six months later.

## Discussion

Caecal diverticulitis is rare in Western countries and has a higher incidence in Oriental populations [1,3]. In Western countries, 85% of all diverticula occur in the descending and sigmoid colon, whereas the incidence of right-sided diverticular disease in Oriental countries can be up to 71%. Solitary caecal diverticulae are believed to be congenital in origin and arise as an out-pouching of the caecum at 6 weeks gestation [1]. As they comprised all layers of the colonic wall, including the muscularis layer, they are therefore designated 'true' diverticula. In contrast, 'false' diverticula are often multiple and consist of herniations of mucosa and submucosa through the circular muscle layer, at the points of penetration of the vasa recta.

The pre-operative diagnosis of right sided colonic diverticulitis is very difficult without radiological imaging [4,5]. Some series have suggested that there are certain clinical features which may help in differentiating the condition from acute appendicitis [3]. These include:

• relatively longer history of right iliac fossa pain

• relative lack of systemic toxic signs despite duration of symptoms

• nausea and vomiting are not common

• symptoms usually begin and remain localised in the right iliac fossa, rather than initially presenting with vague central abdominal pain like appendicitis

However, despite these subtle signs, the condition is usually clinically indistinguishable from acute appendicitis and the correct diagnosis is often made during exploration for suspected appendicitis [4-6].

Because of our strong clinical assumption that our patient had acute appendicitis no pre-operative imaging studies were performed. Both ultrasound (US) [7,8] and computer tomography (CT) [9,10] have been shown to be accurate in diagnosing right side diverticulitis pre-operatively.

The principal US appearances of an inflamed diverticulum are of a round hypo-echoic structure arising from a segment of thickened colonic wall [7,8]. Stronger echoes arising from the structure may represent gas or a faecolith within the diverticular lumen. These features, especially if a normal sonographic appearance of the appendix is found, are highly specific for right-sided diverticulitis. Chou et el [7] reviewed 934 patients with clinically indeterminate right-sided abdominal pain who went on to have abdominal ultrasound. They reported that ultrasound could differentiate between right-sided diverticulitis and acute appendicitis with 100% accuracy. They showed ultrasound to have a sensitivity of 91.3%, a specificity of 99.8% and an overall accuracy of 99.5% in the diagnosis of caecal diverticulitis [7]. False-negative tests were the result of either missing a small diverticulum, suboptimal examination in obese patients or those with abdominal tenderness or the view being obscured by overlying bowel gas [7,8]. Although ultrasound is non-invasive and widely available, operator dependency may limit its accuracy, especially in Western countries where experience of caecal diverticulitis is limited.

Helical CT scanning with intravenous contrast can accurately demonstrate features of acute right sided diverticulitis [9]. Features of caecal diverticulitis on CT are similar to those of left sided diverticulitis and include colonic wall thickening, pericolic fat infiltration, associated abscess formation and extraluminal air denoting perforation. However, these features may also be present with other right sided colonic pathology, such as caecal carcinoma. In approximately 10% of patients diverticulitis is reported to be indistinguishable from carcinoma on CT. Jang et al showed that the presence of an inflamed diverticula and a preserved enhanced pattern of the thickened colonic wall were the two most reliable characteristics to differentiate diverticulitis from caecal carcinoma [9]. In addition a recent study found that visualisation of pericolonic lymph nodes adjacent to the colonic wall lesion was more commonly seen in patients with colonic malignancy [10]. Some authors have suggested that CT scanning is useful in patients with an atypical history for appendicitis, older patients at risk of caecal malignancy and those who have undergone previous appendicectomy [10].

Recently magnetic resonance imaging has been shown to be useful in diagnosis right sided diverticulitis [11]. It may be particularly useful in patients who have equivocal ultrasound features or in those where it is important to avoid ionising radiation, such as young or pregnant patients.

The surgical management of non-perforated caecal diverticulitis is controversial. Table 1 outlines the advantages and disadvantages of each potential management option. If diagnosed confidently pre-operatively, conservative management with intravenous antibiotics, in a similar fashion to the way left-sided diverticulitis is initially managed has the benefit of avoiding laparotomy. If the condition is diagnosed intra-operatively during exploration for appendicitis, conservative management can still be applied after completing the appendicectomy. However, this course of management risks missing an inflammatory carcinoma of the right colon and is more valid in an Asian population where benign pathology is more common than neoplastic disease.

**Table 1:** Advantages and disadvantages of various management approaches in treatment of symptomatic right-sided diverticulitis

Approach, References	Advantages	Disadvantages
**Conservative treatment **[13, 15]	Avoids surgery Applicable for high-risk patients	Only applicable to early stages of diverticulitis High failure rate Disease recurrence
**Diverticulectomy **[1, 15]	Can be performed through appendix incision Low morbidity and mortality	Only suitable for solitary diverticula Under treatment of potential underlying malignancy Not suitable for large inflammatory lesions
**Ileocaecal resection **[2]	Shorter operating time than right hemicolectomy	Under treatment of potential underlying malignancy
**Right hemicolectomy **[1, 13]	Definitive treatment for potential underlying carcinoma	Longer operating time Potentially significant morbidity and mortality Over treats patients with benign pathology

Surgical resection varies from isolated diverticulectomy, ileocaecal resection and right hemicolectomy. Laparoscopic diverticulectomy has also been described in the management of right side diverticulitis [12]. A recent review of 85 patients with caecal diverticulitis, by Fang et al [13], recommend an aggressive resection in treatment of the disease. Less than 40% of their patients were successfully treated conservatively. In the group of patients that had appendicectomy as the only surgical intervention, 29.2% developed recurrent right side diverticulitis and 12.5% required subsequent right hemicolectomy [13].

Other pathology may mimic right side diverticulitis including colonic malignancy, inflammatory Crohn's mass, perforated foreign body reaction or ileocaecal tuberculosis. In our case, the intra-operative findings were suspicious of an underlying carcinoma. In this situation a right hemicolectomy with adequate cancer clearance is the correct surgical procedure. Other indications for aggressive surgical resection include multiple diverticulae or a large caecal phlegmon.

A novel idea, reported by Chui et al [14], to differentiate caecal diverticulitis from caecal carcinoma is the use of intra-operative caecoscopy. During laparotomy, an endoscope is passed through the appendix stump to visualise the caecal mucosa. Although it has only been successfully applied to five patients, its benefits are that if caecal malignancy is excluded the extent of surgical resection can be reduced in uncomplicated cases.

## Conclusion

Caecal diverticulitis, although rare in the Western population, should be considered in the differential diagnosis of patients complaining of right iliac fossa pain. Pre-operative imaging should be considered in patients who present a long history of right iliac fossa pain, lack nausea and vomiting or have an atypical history for acute appendicitis. Ultrasound or computer tomography may reveal the correct diagnosis and has the benefit of avoiding unexpected findings in the operating theatre. The surgical approach should be tailored to the clinical scenario but may include conservative management, diverticulectomy, limited ileocaecal resection or right hemicolectomy. Right hemicolectomy is recommended in patients in whom an underlying carcinoma cannot be confidently excluded and in the context of multiple diverticulae or if a large inflammatory mass is present.

## Competing interests

The author(s) declare that they have no competing interests.

## Authors' contributions

EG performed the literature search and wrote the manuscript; RS performed the surgical procedure, took the clinical photo and helped write the manuscript.

**Consent: **Written consent was obtained from the patient for publication of this case report
